# Real-World Cost-Effectiveness Analysis of Gemcitabine and Cisplatin Compared to Docetaxel and Cisplatin Plus Fluorouracil Induction Chemotherapy in Locoregionally Advanced Nasopharyngeal Carcinoma

**DOI:** 10.3389/fonc.2020.594756

**Published:** 2020-12-23

**Authors:** Jiangping Yang, Jiaqi Han, Jinlan He, Baofeng Duan, Qiheng Gou, Ping Ai, Lei Liu, Yanchu Li, Kexing Ren, Feng Wang, Min Yao, Nianyong Chen

**Affiliations:** ^1^ Department of Head and Neck Oncology and Department of Radiation Oncology, Cancer Center and State Key Laboratory of Biotherapy, West China Hospital, Sichuan University, Chengdu, China; ^2^ Department of Radiation Oncology, University Hospitals Cleveland Medical Center, Case Western Reserve University, Cleveland, OH, United States

**Keywords:** cost-effectiveness, nasopharyngeal carcinoma, induction chemotherapy, gemcitabine, docetaxel, cisplatin, fluorouracil

## Abstract

**Background:**

Addition of gemcitabine and cisplatin (GP) or docetaxel and cisplatin plus fluorouracil (TPF) to concurrent chemoradiotherapy (CCRT) signiﬁcantly improved survival in locoregionally advanced nasopharyngeal carcinoma (NPC). However, an economic evaluation of these regimens remains unknown. The purpose of this study is to compare the cost-effectiveness of GP *versus* TPF regimen in the treatment of locoregionally advanced NPC in China.

**Materials and methods:**

A comprehensive Markov model was developed to evaluate the health and economic outcomes of GP *versus* TPF regimen for patients with locoregionally advanced NPC. Baseline and clinical outcome were derived from 158 patients with newly diagnosed stage III-IVA NPC between 2010 and 2015. We evaluated the quality-adjusted life-years (QALYs), costs, and incremental cost-effectiveness ratios (ICERs) from the perspective of the Chinese healthcare system. One-way sensitive analysis explored the impact of uncertainty in key model parameters on results, and probabilistic uncertainty was assessed through a Monte Carlo probabilistic sensitivity analysis.

**Results:**

GP regimen provided an additional 0.42 QALYs with incremental cost of $3,821.99, resulting in an ICER of $9,099.98 per QALY *versus* TPF regimen at the real-world setting. One-way sensitivity analysis found that the results were most sensitive to the cost and proportion of receiving subsequent treatment in two groups. The probability that GP regimen being cost-effective compared with TPF regimen was 86.9% at a willingness-to-pay (WTP) of $31,008.16 per QALY.

**Conclusion:**

Using real-world data, GP regimen was demonstrated a cost-effective alternative to TFP regimen for patients with locoregionally advanced NPC in China. It provides valuable evidence for clinicians when making treatment decisions to improve the cost-effectiveness of treatment.

## Introduction

Nasopharyngeal carcinoma (NPC) is endemic in southern China and countries in Southeast Asia with around 129,000 new cases and 73,000 disease-related deaths occurred in 2018 (1, 2). More than 70% of patients with NPC are classified as locoregionally advanced disease at diagnosis (3). With the application of intensity-modulated radiotherapy (IMRT) and cisplatin-based concurrent chemoradiotherapy (CCRT), locoregional control of locoregionally advanced NPC has greatly improved (4, 5). However, distant metastasis has emerged as the predominant mode of treatment failure pattern, and it accounts for the cancer-specific mortality among approximately 70% of patients (6, 7).

Induction chemotherapy (IC), given before radiotherapy, offers advantages of satisfactory compliance, early eradication of micro-metastases, and tumor downstaging (8). Varieties of IC regimens have been explored to improve the survival in patients with NPC. Previous randomized phase 3 trials of adding docetaxel and cisplatin plus fluorouracil (TPF) prior to CCRT have significantly prolonged 3-year over survival (OS), failure free survival with acceptable toxicity in patients with locoregionally advanced NPC (9–11). Recently, gemcitabine and cisplatin (GP) has demonstrated its superiority as an induction regimen in locoregionally advanced NPC (12, 13). GP plus CCRT significantly improved 3-year recurrence-free survival and OS among patients with high-risk locoregionally advanced NPC compared to CCRT alone in a multicenter, randomized, phase III trial (13). Based on these encouraging results, sequential GP and TPF regimen followed by CCRT have been both included as preferred choices for IC by the National Comprehensive Cancer Network (NCCN) (14).

Large randomized controlled trials (RCTs) comparing the efficacy of GP with TPF induction regimen are ongoing and no clinical results have been published to date. However, GP regimen has been demonstrated to achieve equivalent efficacy compared to the TPF induction regimen with favorable tolerable adverse events (AEs) in our previous study (15). In situations, where both regimens have survival benefit, increasing medical expenditure and evidence of cost-effectiveness are key factors in clinical decision-making. Further investigation is warranted on whether treatment with GP or TPF regimen improves long-term quality adjusted survival and which regimen is more cost-effective.

Cost-effectiveness analyses for GP or TPF induction chemotherapy in the treatment of NPC have been based on data extracted from RCTs (16–18). Undoubtedly, RCTs provide the best evidence for efficacy, but they may not be the best source of cost data (19). In addition, RCTs are often conducted in highly selected populations and may lack external validity. Therefore, it is difficult to ascertain response in the real-world situation. In this context, we conducted a model-based study to compare the real-world cost-effectiveness of GP and TPF regimen in the setting of locoregionally advanced NPC from the perspective of the Chinese healthcare system.

## Materials and Methods

### Model Structure

**Figure 1:**
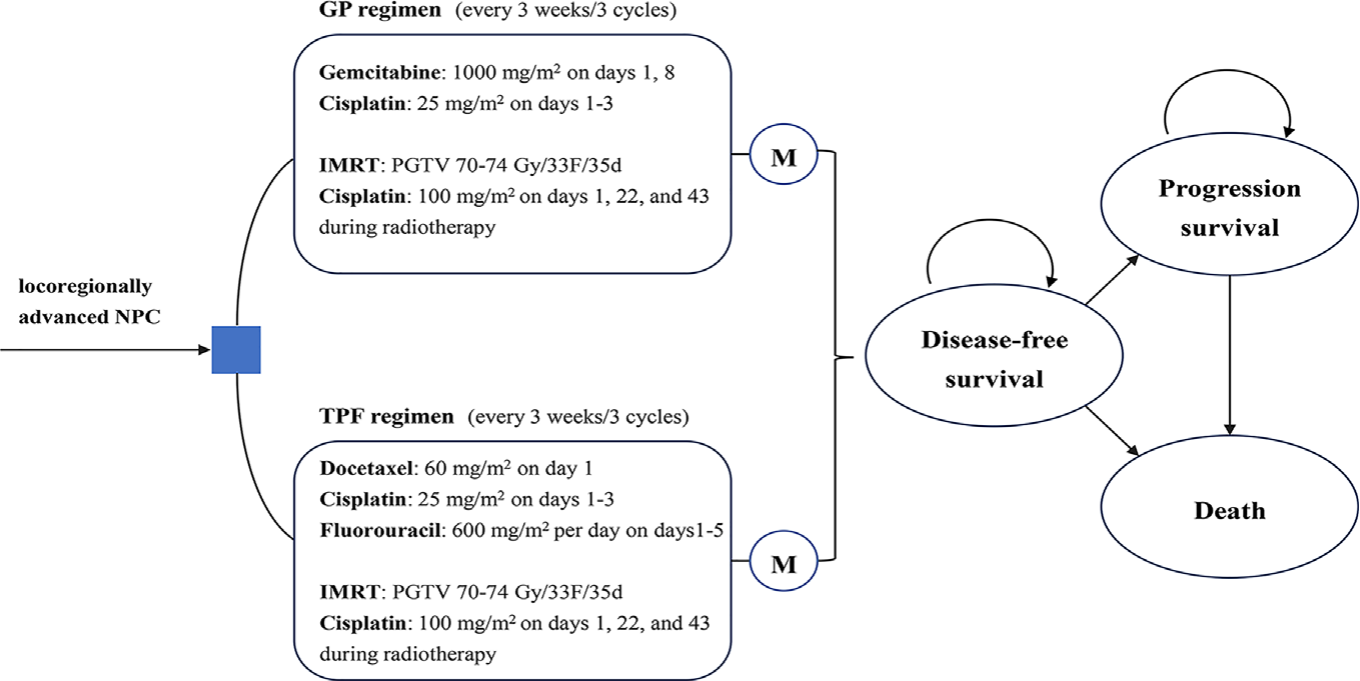
Markov state. Two groups were analyzed in the Markov model: GP group, treated with GP regimen for three cycles followed by CCRT; TFP group, treated with TFP regimen for three cycles followed by CCRT. During each 3-weeks cycle, patients either remained in their assigned health state or progressed to a new health state. CCRT, concurrent chemoradiotherapy; GP, gemcitabine and cisplatin; IMRT, intensity-modulated radiotherapy; M, Markov node; NPC: nasopharyngeal carcinoma; TFP, docetaxel and cisplatin plus fluorouracil.

A comprehensive Markov model was constructed to estimate health and economic outcomes of different treatments for locoregionally advanced NPC patients using real-world clinical data (**Supplementary Figure 1**). As shown in **Figure 1**, the model structure included three exclusive health states to represent different characteristics of locoregionally advanced NPC: disease-free survival (DFS), progressed disease (PD) and death. The Markov cycle length was three weeks, which is consistent with a clinical treatment schedule (15). All patients entered the model in the DFS state and immediately commenced treatment. During each cycle, patients either remained on DFS, progressed to PD or death.

The main outcomes of the study were total costs, life-years (LYs), quality-adjusted life-years (QALYs), and incremental cost-effectiveness ratios (ICERs). A 3% annual discount rate was used for survival and cost estimates. All costs were converted to 2020 US dollars (1 USD = 6.8606 RMB) (20). Three times of the per capita gross domestic product (GDP) in China in 2019 ($31,008.16) was used as a willingness-to-pay (WTP) threshold according to World Health Organization’s criteria (21, 22). The model was performed using the decision analytic software TreeAge Pro 2018 (TreeAge Software, Williamstown, MA).

### Patients and Treatments

Real-world data of 158 locoregionally advanced NPC patients newly diagnosed in West China Hospital Sichuan University were retrospectively analyzed. All patients in our model had stage III–IV disease (except T3-4N0), with Karnofsky performance status scores ≥70; received single-agent cisplatin as the regimen of concurrent chemotherapy and IMRT as the radiotherapy technique. Two treatment options were analyzed in the model: (1) GP group: treated with GP regimen (gemcitabine 1 g/m^2^ on days 1 and 8, cisplatin 25 mg/m^2^ on days 1–3, every three weeks) for three cycles followed by CCRT (cisplatin 100 mg/m^2^ on days 1, 22, and 43; radiotherapy: IMRT); (2) TPF group: treated with TPF regimen (docetaxel 60 mg/m^2^ on day 1, fluorouracil 600 mg/m^2^ on days 1–5, cisplatin 25 mg/m^2^ on days 1–3, every three weeks) for three cycles followed by CCRT. In DFS stage, 71 (44.9%) and 87 (55.1%) patients, respectively, received GP or TPF regimen mainly based on individual patient characteristics (age, gender, performance status, perceived tolerance of chemotherapy, socio-economic status), NCCN guidelines, previous clinical trials results, and willing of patients.

Patients were followed up by magnetic resonance imaging (MRI) scans of the head and neck, chest computed tomography (CT), abdominal sonography, whole blood count, liver and kidney function, thyroid function tests, and Epstein–Barr virus DNA copy number every 3 months during the first 2 years, every 6 months from year 3 to year 5 and annually thereafter. Locoregional recurrence or distant metastasis was diagnosed by biopsy of nasopharyngeal, suspicious lymph node or metastasis lesions. If no biopsy tissue was available, imaging examination, including MRI, CT, whole body bone SPECT imaging, or PET-CT, was allowed to confirm the recurrence or metastasis.

After first-line treatment out to 5 years, a total of 34 (21.5%) patients developed locoregional recurrence or distant metastasis. Based on real-world situation, about 75.0% (9/12) of patients in the GP group and 68.2% (15/22) of patients in the TPF group received further subsequent therapy including chemotherapy and/or radiotherapy for metastatic lesions. We hypothesized that the corresponding proportion of patients in the two groups received subsequent therapy in PD stage in the model. Patients who did not receive subsequent therapy after progression were modeled as receiving only best supportive care (23). The 3-year DFS was 83.1 *vs* 81.6%; the 3-year OS was 94.4 *vs* 92.0% in the GP group, and TPF group, respectively (15).

### Model Transitions and Survival Estimates

Patients moved between health states based on transition probabilities that were calculated from DFS and OS from our previous study (15). First, we extracted data points from Kaplan–Meier survival curves using GetData Graph Digitizer software version 2.26 (http://www.getdata-graph-digitizer.com/) with the method by Hoyle et al. (24). Then, on the basis of the goodness-of-fit examination measured by the adjusted R^2^ statistic, the Weibull survival and Exponential survival model were employed to replicate survival using R software version 3.6.3 (https://www.r-project.org/). The estimated scale and shape parameters, standard errors (SEs), adjusted R^2^ are shown in **Table 1**.

**Table 1:** Model parameters for disease-free survival and overall survival.

Parameters	Survival model	Scale (λ), Mean (SE)	Shape (γ), Mean (SE)	Adjusted R^2^	Correlation Coefficient
DFS					
GP regimen	Weibull	0.00059 (0.00016)	1.48831 (0.07527)	0.95393	−0.97349
TPF regimen	Weibull	0.00256 (0.00063)	1.14471 (0.06796)	0.93703	−0.97115
OS					
GP regimen	Exponential	0.00085 (0.00005)	–	0.81016	−0.95174
TPF regimen	Weibull	0.00097 (0.00033)	1.15449 (0.09365)	0.89176	−0.95184

DFS, disease-free survival; GP, gemcitabine and cisplatin; PD, progress disease; SE, standard error; TPF, docetaxel and cisplatin plus fluorouracil.

### Health-State Utilities

To estimate QALYs, the survival time was adjusted by health-related quality of life. Health utility scores ranged from 0 for death to 1 for perfect health. As no data on quality-of-life was collected in the real-world setting, the utility scores were derived from the other literature for use in the present analysis (25). We assumed 0.76 and 0.57 as the mean utility value for the DFS state and PD state, respectively (**Table 2**).

**Table 2:** Model parameters: baseline values, ranges, and distributions for sensitivity analysis.

Parameters	GP	TPF	Distribution
**DFS cost, $/cycle**			
**Cost of treatment**			
Gemcitabine	559.26 (328.92−902.79)		γ
Docetaxel		764.46 (547.04−1,137.24)	γ
Fluorouracil		148.40 (80.19−200.48)	γ
Cisplatin	13.47 (8.38−16.75)	13.47 (8.38−16.75)	γ
Prophylactic leucocyte		89.86 (71.89−107.83)	γ
Hydration	53.16 (38.91−68.13)	49.36 (32.89−69.90)	γ
Antiemetic drugs	82.78 (66.22−99.33)	82.78 (66.22−99.33)	γ
Hospitalization	115.47 (92.38−138.57)	96.23 (76.98−115.47)	γ
PICC/one time	235.74 (188.60−282.89)	347.24 (277.79−416.69)	Cost: γ Rate: β
Laboratory test	108.20 (86.57−129.85)	119.03 (95.23−142.84)	γ
Imaging examination	201.50 (161.20−241.80)		γ
Radiotherapy	2,624.40 (2,099.52−3,149.28)		γ
Preparation of radiotherapy/one time	1,066.24 (852.99−1,279.48)		γ
**DFS cost, ($)/cycle**			
Subsequent treatment	541.36 (433.09−649.63)	524.08 (419.26−628.89)	γ
Best supportive care	52.53 (42.02−63.04)		γ
**Management of AEs/cycle**			
Grades 1−2	372.42 (124.57−731.95)	345.50 (117.46−708.22)	γ
Grades 3−4	655.52 (399.58−1093.12)	770.69 (296.43−1820.50)	γ
**Cost of follow-up/cycle**			
First 2 years	147.26 (117.81−176.71)		γ
Years 3−5	81.58 (65.27−97.90)		γ
Years 6−10	40.79 (32.63−48.95)		γ
**Utility**			
Utility of DFS	0.76 (0.61−0.91)		β
Utility of PD	0.57 (0.46−0.68)		β
**Other**			
Cost and QALYs discount rate/year	3%		
Willingness to pay	$31,008.16 (3×per capita gross domestic product)		
Exchange rates	$1 = ¥ 6.8606 (2020.1) http://www.pbc.gov.cn/rmyh/108976/109428/index.html		

AE, adverse event; ICER, incremental cost-effectiveness ratio; PICC, peripherally inserted central catheter; QALY, quality-adjusted life-year.

### Cost Estimates

Direct medical costs related to the practice were considered, including patient level drug acquisition, management of treatment-related AEs, radiotherapy, hospitalization, routine follow-up (inpatient and/or outpatient) and subsequent treatments. Other direct costs, including cost of peripherally inserted central catheter, hydration, and prophylactic leucocyte, were also considered as a part of total cost. All costs and events probabilities in our model were obtained from actual clinical practice in West China Hospital Sichuan University (**Table 2**).

For drug acquisition, the actual number of single-use vials and the unit cost per vial were used to calculate the cost of chemotherapy drugs per cycle (**Supplementary Table S1**). For prophylactic leucocyte therapy, granulocyte colony-stimulating factor was administered hypodermic injection at a dose of 5 μg/kg according to NCCN guidelines (14). In our analysis, the most common grade three and four AE was leucopenia, which occurred significantly more often in the TPF group than in the GP group (34.48 *vs* 14.08%, respectively) (15). There was also more neutropenia in the TPF group than in the GP group (24.14 *vs* 14.08%, respectively) (15). We calculated the expected costs of AEs by summing the unit cost of each AE multiplied its probability. And the AEs’ costs were added in the first cycle. **Supplementary Table S2** provides an overview of AE probabilities and costs.

Follow-up cost was directly derived from the real-world patients and found to be independent of types of treatment. Three-monthly cost of $631.10 was applied over the first 2 years of follow-up. From year 3 to 5, a cost of $691.28 was payed for every 6 months. For year 6 and beyond, no data were available, therefore, a cost of $691.28 was assumed annually thereafter.

### Sensitivity Analyses

One-way and probabilistic sensitivity analyses were performed to investigate the impact of varying alternative parametric assumptions on the ICER of GP regimen versus TPF regimen. For costs and events probabilities, the lower and upper limits were based on the actual practical situation whenever available or by assuming ±20% of baseline value if the data was not available. In one-way sensitivity analysis, all relevant parameters were adjusted solely over their defined range and examined the individual effects on ICERs. In probabilistic sensitivity analyses, Monte Carlo simulations with 10,000 replicated random samples were computed, where gamma distribution was assigned to costs, and beta distribution was assigned to clinical probabilities, utility scores and the transition probability. The results were presented as scatter plots and cost-effectiveness acceptability curves to predict the cost-effective possibility of each treatment strategy under different WTP thresholds.

## Results

### Patients Characteristics

From December 2010 to June 2015, a total of 329 newly diagnosed NPC patients were retrieved. And 158 patients were included for analysis, including 71 patients (44.9%) in the GP group and 87 patients (55.1%) in the TPF group, respectively. The baseline characteristics of patients in the model were derived from the relevant data of our previous study (15). Patients’ characteristics, including age, gender, Karnofaky performance status, T classification, N classification, and disease stage, were well balanced between two groups (**Table 3**).

**Table 3:** Simulated patient population and clinical characteristics.

Patient characteristics	All patients (n = 158) No. (%)	GP (n = 71) No. (%)	TPF (n = 87) No. (%)	P
**Age**				0.297
Median, range	46 (19−71)	48 (19−68)	45 (22−71)	
<46	74 (46.8)	30 (42.3)	44 (50.6)	
≥46	84 (53.2)	41 (57.7)	43 (49.4)	
**Gender**				0.420
Male	106 (67.1)	50 (70.4)	56 (64.4)	
Female	52 (32.9)	21 (29.6)	31 (35.6)	
**Karnofsky Performance Status scores**				0.336
90−100	139 (88.0)	61 (85.9)	79 (90.8)	
70−80	19 (12.0)	10 (14.1)	8 (9.2)	
**Tumor stage**				0.843
T1−2	48 (30.4)	21 (29.6)	27 (31.0)	
T3−4	110 (69.6)	50 (70.4)	60 (69.0)	
**Node stage**				0.194
N1	42 (26.6)	15 (21.1)	27 (31.0)	
N2	79 (50.0)	41 (57.7)	38 (43.7)	
N3	37 (23.4)	15 (21.1)	22 (25.3)	
**Disease stage**				0.810
III	55 (34.8)	24 (33.8)	31 (35.6)	
IVA	103 (65.2%)	47 (66.2)	56 (64.4)	

GP, gemcitabine plus cisplatin; TFP, docetaxel, fluorouracil plus cisplatin.

### Base-Case Analysis

Over a 10-year life horizon, although the total cost of GP regimen was higher than that of TPF regimen, the survival benefit of GP regimen was better. Treatment with GP regimen had a total cost of $37,368.55 *versus* $33,546.56 for TPF regimen (**Table 4**). Total LYs for each treatment were 8.10 for GP regimen, 7.43 for TPF regimen. Accounting for quality of life, total QALYs for each treatment were 5.66, 5.24 for GP and TPF regimen, respectively. Therefore, GP regimen achieved an additional 0.42 QALYs with incremental cost of $3,821.99 compared with TPF regimen, resulting in an ICER of $9,099.98 per QALY.

**Table 4:** Summary of base cases results.

	Cost results, $		LY		QALY		ICER	
	Total	Incremental	Total	Incremental	Total	Incremental	per LY	per QALY
Total								
GP	37,368.55	3,821.99	8.10	0.67	5.66	0.42	5,704.46	9,099.98
TPF	33,546.56	–	7.43	–	5.24	–	–	–
DFS state								
GP	18,909.36	–	5.51	0.22	4.19	0.17		
TPF	19,930.34	1,020.98	5.29	–	4.02	–	Dominated	Dominated
PD state								
GP	18,459.19	4,842.97	2.59	0.45	1.47	0.25	10762.16	19,371.88
TPF	13,616.22	–	2.14	–	1.22	–		

ICER, incremental cost-effectiveness ratio; LY, life year; QALY, quality-adjusted life-year.

In DFS stage, GP regimen had greater QALYs (4.19 *vs* 4.02) with lower cost ($18,909.36 *vs* $19,930.34) than did TPF regimen. Therefore, the GP regimen dominated the TPF regimen. In PD stage, GP regimen was more expensive (incremental costs of $4,842.97) and more effective (incremental QALYs of 0.25) than TPF regimen, which resulted in an ICER of $19,371.88 per QALY.

### Sensitivity Analyses

**Figure 2:**
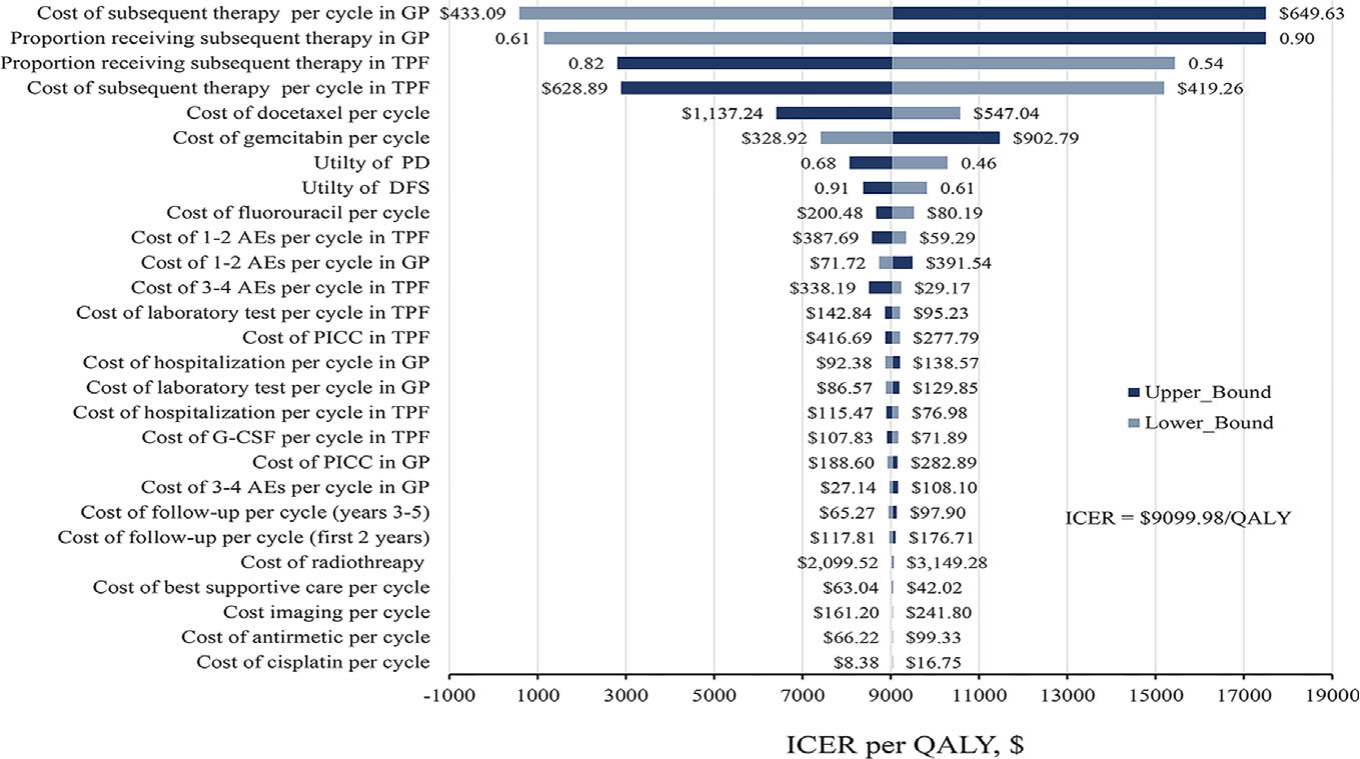
Tornado diagram of one-way sensitivity analysis. The dark blue bar represents the upper bound and light blue represents the lower bound for each variable. AE, adverse events; DFS, disease-free survival; G-CSF, granulocyte colony-stimulating factor; GP, gemcitabine and cisplatin; ICER, incremental cost-effectiveness ratio; PD, progress disease; PICC, peripherally inserted central catheter; QALY, quality-adjusted life-year, TFP, docetaxel and cisplatin plus fluorouracil.

The results of one-way sensitivity analyses were presented in **Figure 2**. The key model drivers were the cost and proportion of receiving subsequent treatment in GP and TPF regimens. Other considerable influential parameters, such as the cost of gemcitabine and docetaxel per cycle, utility of DFS and PD, and cost of fluorouracil per cycle, had mild impact on economic outcomes. However, none of the variables could increase the ICERs above the WTP thresholds.

**Figure 3:**
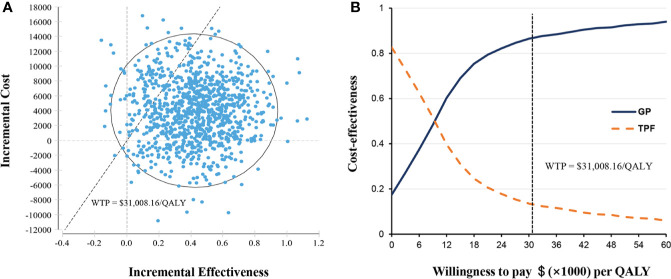
Probabilistic sensitivity analysis. **(A)** Incremental cost-effectiveness scatterplot of 10,000 Monte Carlo simulations shows high probability of cost-effectiveness. **(B)** Cost-effectiveness acceptability curves revealed the results of probabilistic sensitivity analysis under different thresholds of WTP. The dashed line represents the WTP threshold $31,008.16 per QALY. GP, gemcitabine and cisplatin; QALY, quality-adjusted life-years; TFP, docetaxel and cisplatin plus fluorouracil; WTP, willingness-to-pay.

Probabilistic sensitivity analyses showed that the probability of GP regimen being cost-effective was 86.9% at the WTP threshold value of $31,008.16 per QALY compared with TPF regimen (**Figure 3A**). The acceptability curve shows that the relative cost-effectiveness changed with numerical changes in the WTP threshold (**Figure 3B**). When the WTP threshold was one or two times of per capita GDP of China in 2019 ($10,336.05 and $20,672.11, respectively), the probability of the GP regimen achieving cost-effectiveness was about 54.2 and 78.9%, respectively.

### Exploratory on Drug Wastage

To explore the impact of drug wastage on economic effect, we examined drug wastage by calculating chemotherapy drug costs on account of the number of single-use vials used rather than the actual dose administered in clinical practice. When not taking drug wastage into account, the total cost dropped to $37,322.65 in GP regimen, $33,381.69 in TPF regimen, respectively. Therefore, GP regimen provided additional 0.42 QALYs with an incremental cost of $3,940.96, resulting an ICER of $9,383.24 per QALY compared with TPF regimen.

## Discussion

Induction chemotherapy plays an increasingly important role in locoregionally advanced NPC, which helps further improve distant control and overall survival (10, 12, 13). However, the best induction chemotherapy regimen remains to be determined. Furthermore, when new treatment strategies become available, it is essential to assess their potential economic impact before acceptance, especially for countries or regions with limited resources. Our analyses synthesized the real-world data and demonstrated that GP regimen as an induction chemotherapy for patients with locoregionally advanced NPC is more cost-effective compared with TPF at a WTP threshold of $31,008.16 per QALY.

One recent publication performed cost-effectiveness analysis by comparing GP versus PF regimen for first-line treatment of recurrent or metastatic NPC (16), with an ICER of $7,386 per QALY and reported that patients treated with GP regimen achieved an additional incremental QALYs with $5,333 costs compared with PF regimen. Another recent study by Wu et al. estimated that GP was associated with an increase of 2.71 QALYs and $ 7,600 *versus* TPF regimen, resulting in an ICER of $2,804.44 per QALY (18). Both of the two studies suggest GP regimen is an effective, and cost-effective treatment for metastasis or locoregionally advanced NPC compared with PF or TPF regimen. In the latter analysis, Wu et al. created a model based on results from two trials (NCT01245959 and NCT01872962) with different patient populations indirectly comparing GP and TPF regimens (18). The results may not hold true if one population is healthier than another or has access to more efficacious subsequent treatments, and might give rise to the potential for inconsistent and biased analyses (26, 27). In contrast, our study, based on real-world data, directly evaluated the survival and medical costs of two different induction chemotherapy regimens along the follow-up courses to estimate the life expectancy and lifetime costs. Fewer assumptions are required in our analysis, and the cost-effectiveness estimates produce figures much closer to reality. In addition, several non-negligible advantages in the real-world based cost-effective analysis, such as patient selection criteria, treatment patterns and dosing, and the extent of follow-up (28, 29), might represent the experience of patients in actual clinical situation, thus making it valuable for payers to compare the effectiveness of interventions in real practice.

To our knowledge, this is the first real-world cost-effectiveness analysis of GP regimen compared with TPF regimen as induction chemotherapy for patients with locoregionally advanced NPC. Based on a Markov analytic modeling, GP regimen was demonstrated to be more cost-effective than TPF regimen with an ICER of $9,099.98 per QALY from the perspective of Chinese healthcare system. The probabilistic sensitivity analyses suggested a high likelihood (86.9%) that GP regimen would be considered cost-effective at a WTP threshold of $31,008.16 per QALY in China. In DFS stage, GP regimen established total supremacy over TPF regimen because of its lower total cost and superior projected survival benefit. And higher cost of TPF regimen might be the result of the high cost of docetaxel per cycle, followed by a relatively large percentage of patients receiving docetaxel plus cisplatin and fluorouracil who developed more AEs, which are associated with lengthy hospitalization, prolonged treatment course and increased monitoring costs. However, more patients were modeled as receiving subsequent treatment in GP group in PD stage based on the real-word situation, which contributed to the higher total cost compared with TPF regimen.

One-way sensitivity analysis showed that the costs and proportion of subsequent treatment in two groups were the most influential parameters with respect to the robustness of the model. However, the ICERs values were lower than WTP threshold at any of the tested variable lower or upper limits of the parameters. In actual clinical situation, there is inevitable wastage of chemotherapy drugs, and the results had not materially changed without counting the wastage of chemotherapy drugs in our model. Although drugs vials sharing could control drug wastage, it might cause extensive harm to patients. Moreover, it violates the provisions of Centers for Disease Control and Prevention that single-dose or single-use vials should be used for only one patient (30, 31).

Using the clinical and economic data based on our real-world cohorts, this study directly compared the cost-effectiveness of GP and TPF induction chemotherapy in locoregionally advanced NPC, and provided insight into real-world effects. However, there are some limitations in our study. First, the utility values for the calculation of QALYs were extracted from a published study but not data prospectively collected. Sensitivity analysis was performed for the utilities to make sure that the effect of this parameter on long-term results was modest across the two treatment strategies. Second, the results might be limited because of the small sample size from a single institution, which are subject to variation throughout the rest of China. Considering the impact of regional differences on the results, the price range of ±20% was calculated to extend the applicable population and regions of the country. And further multicenter randomized trials with large patient population are needed to confirm our finding. Third, immunotherapy emerges as a promising treatment option for NPC patients in recent years (32–34). However, during the study period of 2010–2015, few patients with advanced NPC received immunotherapy after the first-line standard treatment. Therefore, the effectiveness of immunotherapy in second and subsequent treatment lines as well as detailed real-world cost analysis including costs of anti-PD-1 agents and other procedures remains a subject for further research.

In conclusion, the present study provides valuable real-world evidence that GP regimen was more cost-effective compared with TPF regimen for patients with locoregionally advanced NPC at the WTP threshold of $31,008.16 in China. Further head-to-head clinical trials will provide valuable insight into the optimal induction chemotherapy regimen for NPC.

## Data Availability Statement 

The original contributions presented in the study are included in the article/**Supplementary Material**. Further inquiries can be directed to the corresponding author.

## Author Contributions

JY: software, methodology, formal analysis, writing-original draft. JQH and JLH: software, formal analysis, investigation. BD, QG, and PA: investigation, validation. LL, YL, KR, and FW: supervision and clinical input. MY: investigation, writing—review and editing. NC: conceptualization and study design, writing—review and editing. All authors contributed to the article and approved the submitted version.

## Conflict of Interest

The authors declare that the research was conducted in the absence of any commercial or financial relationships that could be construed as a potential conflict of interest.
