# Supplementary material for: Real-World Cost-Effectiveness Analysis of Gemcitabine and Cisplatin Compared to Docetaxel and Cisplatin Plus Fluorouracil Induction Chemotherapy in Locoregionally Advanced Nasopharyngeal Carcinoma
Source: Front Oncol. 2020 Dec 23;10:594756. doi: 10.3389/fonc.2020.594756 (PMC7786365; doi:10.3389/fonc.2020.594756)
Supplement: Supplementary file 1 [file DataSheet_1.docx]

Supplementary Material

Supplementary Figure and Tables

**Supplementary Figure 1.** Decision-analytic model used in this study.

**Supplementary Table 1.** Treatment regimens and unit costs.

**Supplementary Table 2**. Unit cost and incidence rate of 1 - 4 grade adverse events in actual clinical practice.

**Supplementary Table 3.** CHEERS checklist.

**
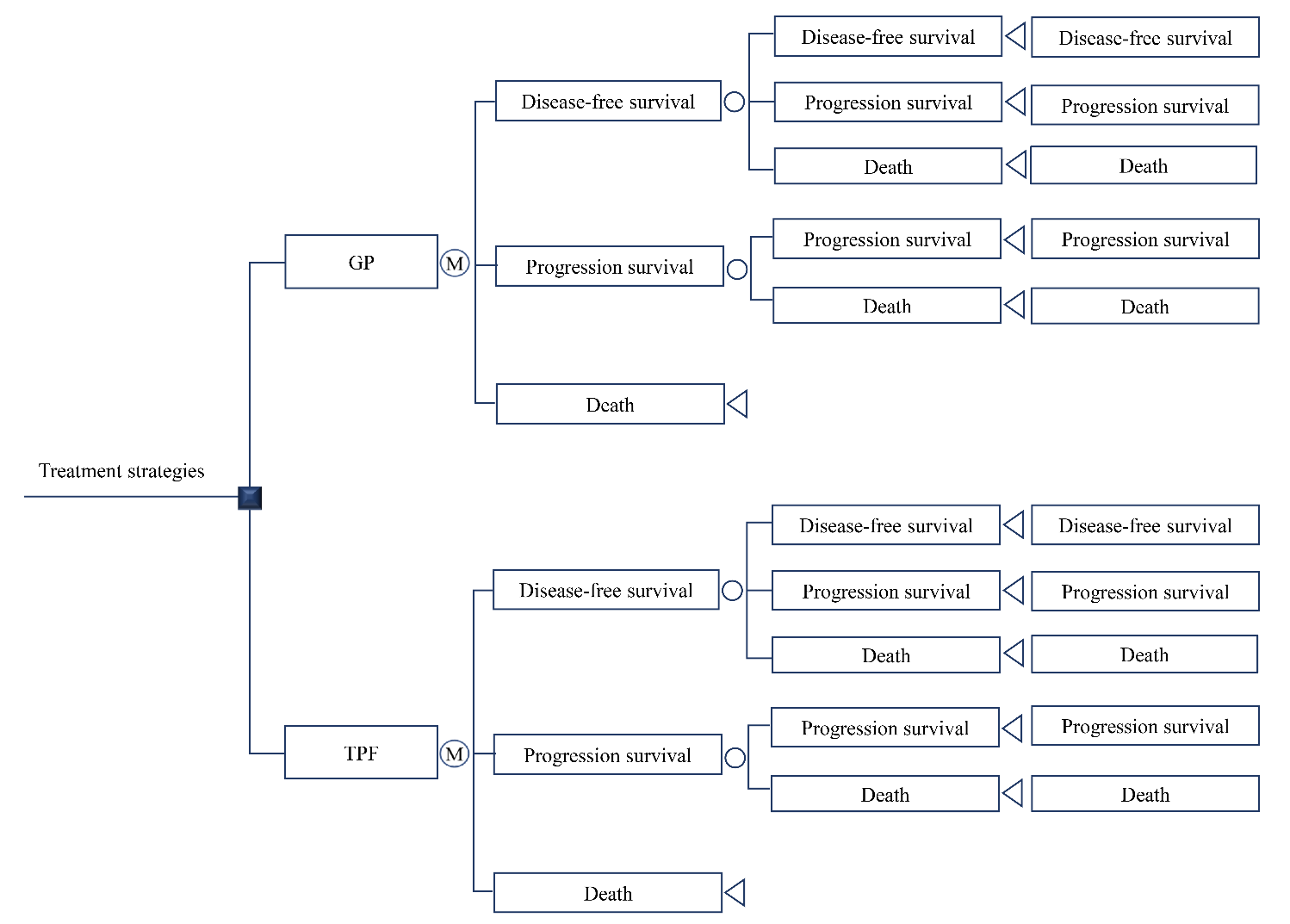
**

**Supplementary Figure 1.** Decision-analytic model used in this study. Two groups were analyzed in the Markov model: GP group, patients with locoregionally advanced NPC treated with GP regimen for three cycles followed by CCRT; TFP group, patients with locoregionally advanced NPC treated with TFP regimen for three cycles followed by CCRT. The Markov model madding up of three health states (disease-free survival, progression survival, and death) was performed. CCRT, concurrent chemoradiotherapy; GP, gemcitabine and cisplatin; NPC, nasopharyngeal carcinoma; TPF, docetaxel and cisplatin plus fluorouracil.

**Supplementary Table 1.** Treatment regimens and unit costs.

| **Treatment** | **Regimen components** | **Dosing schedule per cycle** | **Unit** | **Unit cost ($)** |
| --- | --- | --- | --- | --- |
| **Induction regimen** | | | | |
| **GP** | Gemcitabine | 1000mg/m^2^ (ivgtt on days 1, 8) | 1×200mg vial | 20.56 or 50.16 |
|  | Cisplatin | 25mg/m^2^ (ivgtt on days 1-3) | 1×6ml:30mg vial | 2.79 |
| **TPF** | Docetaxel | 60 mg/m^2^ (ivgtt on day 1) | 1×20mg vial | 136.76 or 189.54 |
|  | Fluorouracil | 600mg/m^2^ (ivgtt on days 1-5) | 1×10ml:250mg vial | 8.02 |
|  | Cisplatin | 25mg/m^2^ (ivgtt on days 1-3) | 1×6ml:30mg vial | 2.79 |
| **CCRT** |  | | | |
| **Chemotherapy** | Cisplatin | 100mg/m^2^ on day 1, 22, and 43 or  25mg/ m^2^/d on days 1-3, 22-24, and 43-45 during radiotherapy | 1×6ml:30mg vial | 2.79 |
| **Radiotherapy** | IMRT | PGTV: 70-74 Gy /33F/35d  PTV60: 60 Gy/33F/35d  PTV54: 54 Gy/33F/35d | 2.12-2.24 Gy | 174.96 |

GP, gemcitabine plus cisplatin; TFP, docetaxel, fluorouracil plus cisplatin; CCRT, concurrent chemoradiotherapy; IMRT, intensity-modulated radiotherapy.

**Supplementary Table 2**. Unit cost and incidence rate of 1 - 4 grade adverse events in actual clinical practice.

|  | Baseline value | Range | | Distribution (parameters) |
| --- | --- | --- | --- | --- |
|  |  | Minimum | Maximum |  |
| **Management of AEs ($)** |  |  |  |  |
| **TPF Group** |  |  |  |  |
| **Grade 1-2** |  |  |  |  |
| Leucopenia | 47.13 | 7.52 | 116.04 | Gamma (2.898, 0.061) |
| Neutropenia | 58.59 | 7.52 | 116.04 | Gamma (4.479, 0.076) |
| Nausea | 26.74 | 16.56 | 51.33 | Gamma (9.088, 0.340) |
| Vomiting | 28.56 | 16.56 | 51.33 | Gamma (10.368, 0.363) |
| Liver | 105.79 | 26.48 | 162.00 | Gamma (9.364, 0.089) |
| Diarrhea | 47.43 | 30.38 | 107.68 | Gamma (5.785,0.122) |
| Mucositis | 31.26 | 12.44 | 103.80 | Gamma (1.799, 0.058) |
| **Grade 3-4** |  |  |  |  |
| Leucopenia | 108.49 | 15.05 | 301.32 | Gamma (2.207,0.020) |
| Neutropenia | 149.37 | 15.05 | 710.25 | Gamma (0.709, 0.005) |
| Nausea | 76.19 | 38.38 | 124.88 | Gamma (11.922, 0.156) |
| Vomiting | 96.61 | 57.50 | 122.58 | Gamma (33.863, 0.351) |
| Liver | 144.11 | 63.64 | 224.58 | Gamma (12.321, 0.855) |
| Diarrhea | 141.38 | 80.90 | 201.86 | Gamma (20.993, 0.148) |
| Mucositis | 54.54 | 25.91 | 135.03 | Gamma (3.839, 0.070) |
| **GP group** |  |  |  |  |
| **Grade 1-2** |  |  |  |  |
| Leucopenia | 43.22 | 12.68 | 93.34 | Gamma (4.412, 0.102) |
| Neutropenia | 44.14 | 12.68 | 119.85 | Gamma (2.607, 0.059) |
| Nausea | 39.11 | 23.03 | 61.04 | Gamma (16.269, 0.416) |
| Vomiting | 40.43 | 23.03 | 61.04 | Gamma (17.385, 0.430) |
| Liver | 126.17 | 28.84 | 229.72 | Gamma (6.062, 0.048) |
| Diarrhea | 52.50 | 13.15 | 100.23 | Gamma (5.585, 0.106) |
| Mucositis | 26.85 | 11.16 | 66.73 | Gamma (3.587, 0.134) |
| **Grade 3-4** |  |  |  |  |
| Leucopenia | 69.76 | 13.07 | 127.11 | Gamma (5.750, 0.082) |
| Neutropenia | 71.04 | 37.03 | 127.11 | Gamma (9.557, 0.135) |
| Nausea | 86.99 | 59.92 | 120.18 | Gamma (32.022, 0.368) |
| Vomiting | 97.14 | 72.53 | 132.29 | Gamma (40.602, 0.418) |
| Liver | 168.03 | 134.42 | 201.63 | Gamma (96.046, 0.572) |
| Diarrhea | 77.61 | 62.09 | 93.13 | Gamma (96.065, 1.238) |
| Mucositis | 84.95 | 20.52 | 291.67 | Gamma (1.508, 0.018) |
| **Probabilities of AEs** |  |  |  |  |
| **TPF group** |  |  |  |  |
| **Grade 1-2** |  |  |  |  |
| Leucopenia | 54.02% | 43.22% | 64.82% | Beta (0.540, 0.460) |
| Neutropenia | 63.22% | 50.58% | 75.86% | Beta (0.632, 0.368) |
| Nausea | 74.71% | 59.77% | 89.65% | Beta (0.747, 0.253) |
| Vomiting | 57.47% | 45.98% | 68.96% | Beta (0.575, 0.425) |
| Liver | 58.62% | 46.90% | 70.34% | Beta (0.586, 0.414) |
| Diarrhea | 13.80% | 11.04% | 16.56% | Beta (0.138, 0.862) |
| Mucositis | 71.26% | 57.01% | 85.51% | Beta (0.713, 0.287) |
| **Grade 3-4** |  |  |  |  |
| Leucopenia | 34.48% | 27.58% | 41.38% | Beta (0.345, 0.655) |
| Neutropenia | 24.14% | 19.31% | 28.97% | Beta (0.241, 0.759) |
| Nausea | 6.90% | 5.52% | 8.28% | Beta (0.069, 0.931) |
| Vomiting | 17.24% | 13.79% | 20.69% | Beta (0.172, 0.828) |
| Liver | 2.30% | 1.84% | 2.76% | Beta (0.023, 0.977) |
| Diarrhea | 2.30% | 1.84% | 2.76% | Beta (0.023, 0.977) |
| Mucositis | 17.24% | 13.79% | 20.69% | Beta (0.172, 0.828) |
| **GP group** |  |  |  |  |
| **Grade 1-2** |  |  |  |  |
| Leucopenia | 61.97% | 49.58% | 74.36% | Beta (0.620, 0.380) |
| Neutropenia | 56.34% | 45.07% | 67.61% | Beta (0.563, 0.437) |
| Nausea | 76.06% | 60.85% | 91.27% | Beta (0.761, 0.239) |
| Vomiting | 63.38% | 50.70% | 76.06% | Beta (0.634, 0.366) |
| Liver | 46.48% | 37.18% | 55.78% | Beta (0.465, 0.535) |
| Diarrhea | 33.80% | 27.04% | 40.56% | Beta (0.338, 0.662) |
| Mucositis | 60.56% | 48.45% | 72.67% | Beta (0.606, 0.394) |
| **Grade 3-4** |  |  |  |  |
| Leucopenia | 14.08% | 11.26% | 16.90% | Beta (0.141, 0.859) |
| Neutropenia | 14.08% | 11.26% | 16.90% | Beta (0.141, 0.859) |
| Nausea | 7.04% | 5.63% | 8.45% | Beta (0.070, 0.930) |
| Vomiting | 14.08% | 11.26% | 16.90% | Beta (0.141, 0.859) |
| Liver | 1.41% | 1.13% | 1.69% | Beta (0.014, 0.986) |
| Diarrhea | 1.41% | 1.13% | 1.69% | Beta (0.014, 0.986) |
| Mucositis | 14.08% | 11.26% | 16.90% | Beta (0.141, 0.859) |

AE, adverse events; GP, gemcitabine and cisplatin; TFP, docetaxel and cisplatin plus fluorouracil.

**Supplementary Table 3.** CHEERS checklist

| Section/item | Item No | Recommendation | Reported? |
| --- | --- | --- | --- |
| Title and abstract | | | |
| Title | 1 | Identify the study as an economic evaluation or use more specific terms such as “cost-effectiveness analysis”, and describe the interventions compared. | Yes |
| Abstract | 2 | Provide a structured summary of objectives, perspective, setting, methods (including study design and inputs), results (including base case and uncertainty analyses), and conclusions. | Yes |
| Introduction | | | |
| Background and objectives | 3 | Provide an explicit statement of the broader context for the study. Present the study question and its relevance for health policy or practice decisions. | Yes |
| Methods | | | |
| Target population and subgroups | 4 | Describe characteristics of the base case population and subgroups analyzed, including why they were chosen. | Yes |
| Setting and location | 5 | State relevant aspects of the system(s) in which the decision(s) need(s) to be made | Yes |
| Study perspective | 6 | Describe the perspective of the study and relate this to the costs being evaluated. | Yes |
| Comparators | 7 | Describe the interventions or strategies being compared and state why they were chosen. | Yes |
| Time horizon | 8 | State the time horizon(s) over which costs and consequences are being evaluated and say why appropriate. | Yes |
| Discount rate | 9 | Report the choice of discount rate(s) used for costs and outcomes and say why appropriate. | Yes |
| Choice of health outcomes | 10 | Describe what outcomes were used as the measure(s) of benefit in the evaluation and their relevance for the type of analysis performed. | Yes |
| Measurement of effectiveness | 11a | Single study-based estimates: Describe fully the design features of the single effectiveness study and why the single study was a sufficient source of clinical effectiveness data. | Yes |
|  | 11b | Synthesis-based estimates: Describe fully the methods used for identification of included studies and synthesis of clinical effectiveness data. | N/A |
| Measurement and valuation of preference based outcomes | 12 | If applicable, describe the population and methods used to elicit preferences for outcomes. | N/A |
| Estimating resources and costs | 13a | Single study-based economic evaluation: Describe approaches used to estimate resource use associated with the alternative interventions. Describe primary or secondary research methods for valuing each resource item in terms of its unit cost. Describe any adjustments made to approximate to opportunity costs. | N/A |
|  | 13b | Model-based economic evaluation: Describe approaches and data sources used to estimate resource use associated with model health states. Describe primary or secondary research methods for valuing each resource item in terms of its unit cost. Describe any adjustments made to approximate to opportunity costs. | Yes |
| Currency, price date, and conversion | 14 | Report the dates of the estimated resource quantities and unit costs. Describe methods for adjusting estimated unit costs to the year of reported costs if necessary. Describe methods for converting costs into a common currency base and the exchange rate. | Yes |
| Choice of model | 15 | Describe and give reasons for the specific type of decision-analytical model used. Providing a figure to show model structure is strongly recommended. | Yes |
| Assumptions | 16 | Describe all structural or other assumptions underpinning the decision-analytical model. | Yes |
| Analytical methods | 17 | Describe all analytical methods supporting the evaluation. This could include methods for dealing with skewed, missing, or censored data; extrapolation methods;  Yes  methods for pooling data; approaches to validate or make adjustments (such as half cycle corrections) to a model; and methods for handling population heterogeneity and uncertainty. | Yes |
| Results | | | |
| Study parameters | 18 | Report the values, ranges, references, and, if used, probability distributions for all parameters. Report reasons or sources for distributions used to represent uncertainty where appropriate. Providing a table to show the input values is strongly recommended. | Yes |
| Incremental costs and outcomes | 19 | For each intervention, report mean values for the main categories of estimated costs and outcomes of interest, as well as mean differences between the comparator groups. If applicable, report incremental cost-effectiveness ratios. | Yes |
| Characterizing uncertainty | 20a | Single study-based economic evaluation: Describe the effects of sampling uncertainty for the estimated incremental cost and incremental effectiveness parameters, together with the impact of methodological assumptions (such as discount rate, study perspective). | N/A |
|  | 20b | Model-based economic evaluation: Describe the effects on the results of uncertainty for all input parameters, and uncertainty related to the structure of the model and assumptions. | Yes |
| Characterizing heterogeneity | 21 | If applicable, report differences in costs, outcomes, or cost-effectiveness that can be explained by variations between subgroups of patients with different baseline characteristics or other observed variability in effects that are not reducible by more information. | N/A |
| Discussion | | | |
| Study findings, limitations, generalizability, and current knowledge | 22 | Summarize key study findings and describe how they support the conclusions reached. Discuss limitations and the generalizability of the findings and how the findings fit with current knowledge. | Yes |
| Other | | | |
| Source of funding | 23 | Describe how the study was funded and the role of the funder in the identification, design, conduct, and reporting of the analysis. Describe other non-monetary sources of support. | Yes |
| Conflicts of interest | 24 | Describe any potential for conflict of interest of study contributors in accordance with journal policy. In the absence of a journal policy, we recommend authors comply with International Committee of Medical Journal Editors recommendations. | Yes |
